# Sherborn’s foraminiferal studies and their influence on the collections at the Natural History Museum, London

**DOI:** 10.3897/zookeys.550.9863

**Published:** 2016-01-07

**Authors:** C. Giles Miller

**Affiliations:** 1 Department of Earth Sciences, The Natural History Museum, London. SW7 5BD

**Keywords:** Foraminifera, C. D. Sherborn, Fortescue William Millett, T. Rupert Jones, Edward Heron-Allen, A. W. Rowe, C. P. Chatwin, T. H. Withers, Natural History Museum, Chalk, London Clay, Kimmeridge Clay

## Abstract

Sherborn’s work on the Foraminifera clearly provided the initial spark to compile the major indexes for which he is famous. Contact and help from famous early micropalaeontologists such as T. Rupert Jones and Fortescue William Millett led Sherborn to produce his *Bibliography of Foraminifera* and subsequently a two-part *Index of Foraminiferal Genera and Species*. Edward Heron-Allen, whose mentor was Millett, was subsequently inspired by the bibliography to attempt to acquire every publication listed. This remarkable collection of literature was donated to the British Museum (Natural History) in 1926 along with the foraminiferal collections Heron-Allen had mainly purchased from early micropalaeontologists. This donation forms the backbone of the current NHM micropalaeontological collections. The NHM collections contain a relatively small amount of foraminiferal material published by Sherborn from the London Clay, Kimmeridge Clay and Speeton Clay. Another smaller collection reflects his longer-term interest in the British Chalk following regular fieldwork with A. W. Rowe. Other collections relating to Sherborn’s early published work, particularly with T. R. Jones, are not present in the collections but these collections may have been sold or deposited elsewhere by his co-workers.

## Introduction

Whilst Sherborn is best known for his *Index Animalium*, his scientific career began with work on Foraminifera and Ostracoda. Foraminifera are single celled organisms that secrete amazingly diverse microscopic shells or tests of mainly calcite. Occasionally they use available ocean bottom sediment to create their tests. Ostracoda are microscopic bivalved crustaceans common in most aquatic environments and found through most of the fossil record. This paper aims to provide details of Sherborn’s relationships to early micropalaeontological workers, to summarise his work on the Foraminifera and to investigate how this is reflected by the collections currently held at the Natural History Museum, London. For convenience in this paper, the title “Natural History Museum” is used throughout even where the original name of the institution was The British Museum (Natural History).

### The Natural History Museum foraminiferal collections

Roughly half of the Natural History Museum’s microfossil collection of approximately 550,000 slides represent examples of foraminifera with the remainder including ostracods, palynomorphs, calcareous nannofossils, radiolarians and conodonts. The museum’s micropalaeontological collection is built around the donation in 1926 of a remarkable collection of foraminiferal books and slides assembled by Edward Heron-Allen mainly during the early 20^th^ century and subsequently much added to (Hodgkinson 1989, Whittaker 2013). These collections contain fossil and mainly Recent Foraminifera and were originally part of the Zoology Department collections. They were subsequently moved to The Geology Department, part of which later became The Palaeontology Department that later formed part of the current Earth Science Department. A full history of the NHM micropalaeontological collections and their custodians has yet to be written but some historical details of Heron-Allen’s collection of Foraminifera and its acquisition have been provided by Hodgkinson and Whittaker (2003) and other papers such as Adams et al. (1980) give an overview of all type material deposited by 1980. Details of most of the type and figured part of the collection can be found on-line on the museum’s web site (http://data.nhm.ac.uk/).

### Sherborn and T. Rupert Jones

T. Rupert Jones (1819–1911) was a London surgeon who became interested in palaeontology and particularly Foraminifera and Ostracoda. He was later Professor of Geology at the Royal Military College in Sandhurst, Fellow of the Geological Society and was elected Fellow of the Royal Society in 1872 (Siveter and Lord 2004). Evenhuis (2016), Shindler (2016) and Siveter and Lord (2004) describe how in retirement, T. R. Jones employed Sherborn to help illustrate and complete some works on the Foraminifera leading Sherborn to The Department of Geology at the new Natural History Museum at South Kensington where he made contact with several members of staff in The Geology Department. Sherborn claims that he was one of the first dozen visitors through the doors at the new museum when it opened in 1881 and shortly afterwards was employed by The Geology Department to mount specimens (Shindler 2016). Sherborn and T. R. Jones jointly published three papers; two on Foraminifera (Jones and Sherborn 1886, 1887) and another a large monograph on the Ostracoda (Jones and Sherborn 1889).

### Sherborn and Millett

Fortescue William Millett (1833–1915) was one of the leading micropalaeontologists of the 19^th^ Century working mainly on Recent Foraminifera (Hodgkinson 2006, Hart et al. 2011). Although he was born in Cornwall, SW England and retired there, he spent over 30 years in London where he was one of the founder members of the Quekett Microscopical Club in 1865. This is presumably where he came into contact with Sherborn although this is not recorded. Millett had an encyclopaedic knowledge of the Foraminifera and a very good library (Hodgkinson 2006). Sherborn’s acknowledgement of Millett in the 1893 *Index of Genera and Species* is stated in a prefatory note dated October 1893 Millett’s ‘knowledge of literature of the subject is remarkable and peculiar’ (Sherborn 1893, un-numbered page prior to p. 1). Sherborn wrote a short obituary of Millett (Sherborn 1915), which was expanded by Hodgkinson (2006) to provide an in-depth biography of Millett including listing Millett’s 13 papers on the Foraminifera.

Documents rescued by Edward Heron-Allen from Millett’s house after his death suggest that Millett had a strong connection with T. R. Jones who often passed him collections to study (Hodgkinson 2006) and both Sherborn and Millett are acknowledged in T. R Jones’s introduction to the foraminiferal part of the Crag Monograph of East Anglia (Jones 1895).

### Sherborn and Heron-Allen

**Figure 1.**
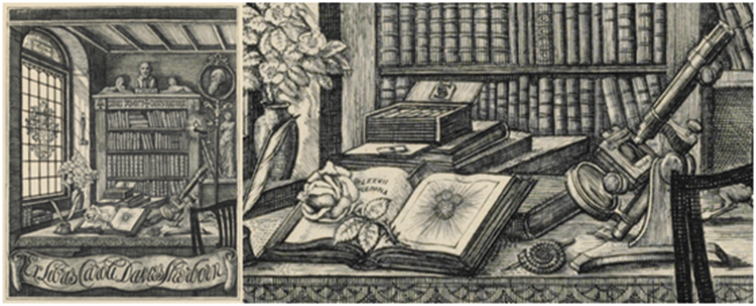
C. D. Sherborn bookplate acquired by Heron-Allen and pasted into the first couple of pages of Heron-Allen’s copy of Sherborn (1888b). Heron-Allen wrote ‘The original figure *Globigerina bulloides* is 23.5cm in height, is quite accurately reproduced.’

We can only assume that Edward Heron-Allen (1861–1943) met Sherborn because Heron-Allen was also greatly influenced by his mentor Millett and was also a member of the Quekett Microscopical Club. The polymathic Heron-Allen had become fascinated by the Foraminifera aged 14 but only started serious study of them relatively late in life. He made it an aim to acquire all of the early works on the Foraminifera listed in Sherborn’s 1888 *Bibliography of Foraminifera* (Jones 2005). Several copies of this 1888 *Bibliography* are present in the Heron-Allen Library at the NHM. Heron-Allen had his personal copy rebound and annotated its margins with references to books in his personal library that he later donated to the British Museum (Natural History). Attached into the front of the same book is C. D. Sherborn’s bookplate (Fig. 1) designed and engraved by Sherborn’s father C. W. Sherborn that illustrates a bust of Shakespeare and a profile portrait of Darwin (Jones 2005). Heron-Allen, a prolific gatherer of related materials, often pasted articles or letters or annotated them but it is not clear how he acquired this particular item. Next to the book plate he left the following handwritten note:

‘Symbolic book plate of the author designed and engraved by his father C. W. Sherborn, the most notable book plate engraver of the XIXth century. NB the miniature reproduction of Plate 77 of H. B. Brady’s Report on the Foraminifera of the Challenger Expedition, London 1884. The original figure *Globigerina bulloides* is 23.5cm in height, is quite accurately reproduced.’

The annotations in Heron-Allen’s copy of the 1888 index suggest that Heron-Allen came close to his aim of acquiring all the references listed in the bibliography as about 80 per cent are accounted for. Whether Heron-Allen and Sherborn ever met is not known for certain. What is certain is that the index guided Heron-Allen to accumulate the amazing collection of foraminiferal books and references that forms the backbone of the NHM Micropalaeontology Sectional Library that now bears the name ‘The Heron-Allen Library’. The library, augmented with Heron-Allen’s annotations and attached documentation is a unique and unrivalled resource for anyone wishing to study the Foraminifera (Whittaker 2013).

### Sherborn’s research on the Foraminifera

Shindler (2016) and Evenhuis (2016) provide a more detailed history of the production of the bibliography that was started in 1886 and published in 1888. While preparing the *Bibliography* he also published on the foraminifera from the London Clay of Piccadilly, London (Sherborn and Chapman 1886) and the Jurassic of England (Jones and Sherborn 1886). He continued his collaboration with T. R. Jones with a publication on the variability in cristellarian Foraminifera (Jones and Sherborn 1887) and their collaboration culminated with the publication of a monograph on the Tertiary Entomostraca (ostracods) of England (Jones and Sherborn 1889). Just before the publication of the *Bibliography* (Sherborn 1888b) he published a savage review of the American Anthony Woodward’s foraminiferal bibliography (Sherborn 1888a) and this almost compromised the publication of his own bibliography (Shindler 2016, Taylor 2016). He also published a short note on the foraminiferan *Webbina irregularis* (d’Orb) from the Oxford Clay at Weymouth (Sherborn 1888c), by way of a comment on the collection of R. Formby Esquire of Bath. An additional note on the Foraminifera of the London Clay from the Drainage Works in Piccadilly, London (Sherborn and Chapman 1889) was published in the same year as a paper on the London Clay from Sheppey (Chapman and Sherborn 1889). Two papers were written in collaboration with H. W. Burrows (Burrows et al. 1890) on the Foraminifera of the Red Chalk of Yorkshire, Norfolk and Lincolnshire and shortly afterwards on the London Clay from Cannon Street rail bridge (Sherborn and Burrows 1891, Fig. 2).

**Figure 2.**
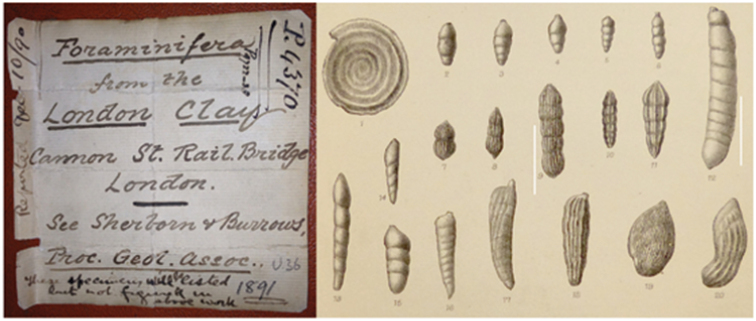
Handwritten note with the collections referring to slides donated by C. D. Sherborn from excavation to widen the Cannon Street Rail Bridge. Representative illustration of Lias Foraminifera from Northamptonshire from Crick and Sherborn (1891, part of fig. 1)

**Figure 3.**
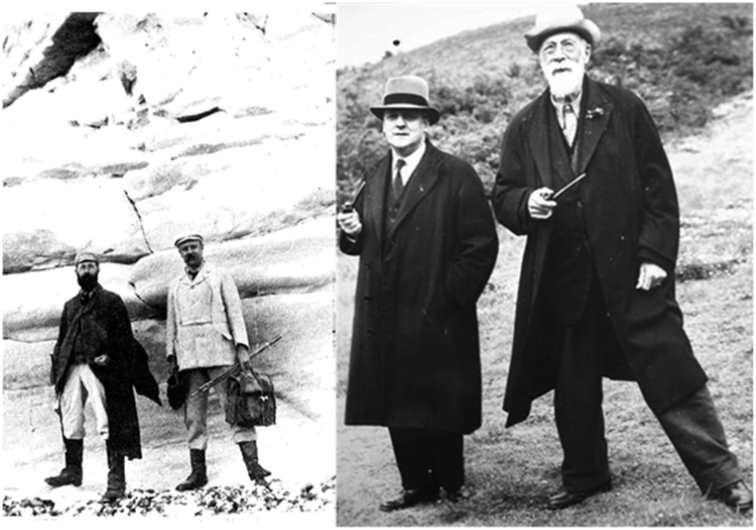
A. Sherborn (left) and A. W. Rowe (right) doing field work on an outcrop of chalk reproduced from Hart and Bailey (2013) with permission from Natural History Museum. B. Sherborn (right) and C. P. Chatwin (left) during fieldwork on the British Chalk.

There followed two publications with Walter Drawbridge Crick (1857–1903) on the Liassic Foraminifera from Northamptonshire (Crick and Sherborn 1891, 1892). To accompany his 1888 *Bibliography of the Foraminifera*, Sherborn then compiled his *Index of Foraminiferal Genera and Species* in two parts (Sherborn 1893, 1896). At around the same time, he published on ostracods from the Gault at Folkestone (Chapman and Sherborn 1893) remarking that these had been recovered during the production of a monograph on the Foraminifera. Sherborn was a lifelong friend and field companion of A. W. Rowe (1858–1926; Fig. 3A) who published a string of papers on the zonation of the English Chalk including one in 1930 that was finished after his death by Sherborn and T. H. Withers (1883–1953) (Gale and Cleevely 1989, Hart and Bailey 2013). This paper includes listings of foraminiferal species but not illustrations or discussions on Foraminifera. In C. S. Carter’s Presidential address to the Lincolnshire Naturalists’ Union for 1928 (Carter 1929) he states that Rowe and Sherborn worked closely together on the Chalk of Lincolnshire, assisted by local amateurs. Gale and Cleevely (1989) provide details of the relationship between Rowe and Sherborn including examples of work Sherborn was responsible for and various anecdotes that suggest that Rowe used Sherborn as a dogsbody. Sherborn is also known to have carried out fieldwork on the Chalk with Charles P. Chatwin (1887–1971, Fig. 3B) who published on the Foraminifera of the Chalk from Oxfordshire and Berkshire with T. H. Withers (Chatwin and Withers 1908). This published collection is held at The Natural History Museum (NHMUK PM P 8762-8786; P 8711-8714). Chatwin was at the time an attendant at the museum and went on to become Librarian at the Geological Society (1913–19), lecturer in Palaeontology at the University of Liverpool (1919–20) and worked at the Geological Survey from 1920 to 1941 (Andrew Morrison, pers. com.).

### Sherborn’s foraminiferal collections at The Natural History Museum

**Figure 4.**
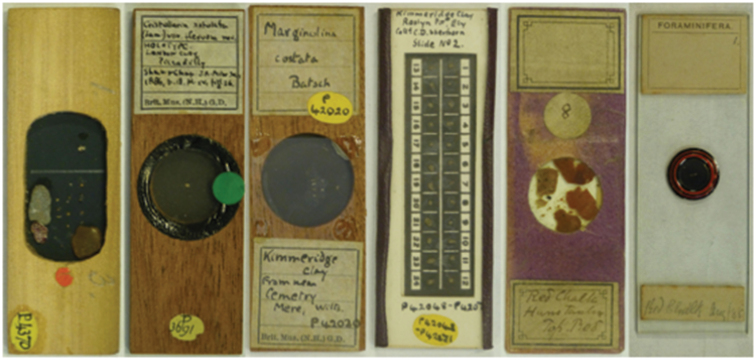
Examples of Sherborn slides from the Natural History Museum collections.

The collections are relatively modest compared to the number of Sherborn’s publications. It may be that the collections were deposited elsewhere or sold if Sherborn was not the first author. The registers show that Sherborn sold material to the British Museum in 1886 and later donated material in 1890. We know that many of T. R. Jones’s collections were sold after his death (Siveter and Lord 2004). Collections not present include material from the London Clay of Sheppey (Chapman and Sherborn 1889) and the material from the Northamptonshire Lias published by Crick et al. (1891, 1892). Examples of Sherborn slides in The Natural History Museum collection are shown (Fig. 4) in the hope that similar slides might be recognised in other collections.

Four discrete collections remain at the Natural History Museum:

1. 21 slides from an excavation of the London Clay at Piccadilly (NHMUK PM P 3669-3726) were purchased from Sherborn in October 1886 and represent the material relating to Sherborn and Chapman (1886).
2. 18 slides from the works to widen the Cannon Street Rail Bridge (NHMUK PM P 4370, 9722-9738) were presented by Sherborn in 1890 and relate to the publication Sherborn and Burrows (1891).
3. 25 slides from several Kimmeridgian, Jurassic sites at Roslyn Pit, Ely, Cambridgeshire, Gillingham Brick Works, Dorset and from near the cemetery at Mere, Wiltshire (NHMUK PM P 42004-42119, 42180-42193). 2 other slides (NHMUK PM P 33316-33317) are from Ely and marked as collected and presented by Sherborn c. 1899. They were published by Ovey (1938).
4. 14 slides from the Red Chalk at Hunstanton and from the Speeton Clay are unregistered but the slide labels indicate that they were prepared by the Rev. G. Bailey. They may relate in part to the publication of Burrows et al. (1890).

## Concluding statements

Sherborn’s foraminiferal collections at the NHM are relatively modest in size and some key collections that he published on are not present. His collections and work on the Foraminifera cannot be considered to be particularly ground breaking. In contrast, the production of the Bibliography and Indexes had a profound effect on Edward Heron-Allen whose subsequent donation of literature and collection forms the backbone of the current Natural History Museum micropalaeontology collection. Publication of the foraminiferal bibliography and indexes also had a profound effect on Sherborn who went on to publish his *Index Animalium*. It seems that later in his life Sherborn continued to encourage workers such as A. W. Rowe, T. H. Withers and C. P. Chatwin to work on foraminifera from the British Chalk, some collections of which are also housed at the Natural History Museum.

## Footnote

The collections at The Natural History Museum continue to be influenced by a modern day C. D. Sherborn. Dr John Williams has been compiling an index of all papers relating to Palaeopalynology. His index of currently stands at 25,502 items, all cross referenced by a vast card index (Riding et al. 2012).
